# AAV-mediated NT-3 overexpression protects cochleae against noise-induced synaptopathy

**DOI:** 10.1038/s41434-018-0012-0

**Published:** 2018-03-13

**Authors:** Hengchao Chen, Yazhi Xing, Li Xia, Zhengnong Chen, Shankai Yin, Jian Wang

**Affiliations:** 1 Otolaryngology Research Institute, 6th Affiliated Hospital, Shanghai Jiao Tong University, Shanghai, China; 2 School of Human Communication Disorders, Dalhousie University, Halifax, NS Canada

## Abstract

The synapse between inner hair cells (IHCs) and type I spiral ganglion neurons (SGNs) has been identified as a sensitive structure to noise-induced damage in the mammalian cochlea. Since this synapse provides the major information pathway from the cochlea to the auditory brain, it is important to maintain its integrity. Neurotrophin-3 (NT-3) has been known to play an important role in the development and the functional maintenance of this synapse. Application of exogenous NT-3, or overexpression of this gene in a transgenic animal model, have shown the value to protect this synapse from noise-induced damage. In the present study, NT-3 overexpression was induced by cochlear gene transfection before noise exposure via the use of an adeno-associated viral (AAV) vector. We found that such an overexpression provided a significant synaptic protection against a noise exposure that caused massive damage to the synapses, likely due to it promoting the repair of the synapse after the initial damage.

## Introduction

Recent studies in animal models have shown that a brief noise exposure of moderate level can produce massive damage around the synapses between inner hair cells (IHCs) and type I spiral ganglion neurons (SGNs) without causing a permanent threshold shift (PTS) [1–4], which is the typical criterion defining noise-induced hearing loss (NIHL). The damage to the synapses abolishes the function of the corresponding SGNs, which would undergo a degenerative death if their synapses to IHCs are not re-established. Furthermore, the SGNs connecting IHCs via residual and re-established synapses may have functional deficits [1, 5, 6]. Clinical evidence suggests that such synaptic damage is likely to occur in human subjects after moderate noise exposures that are considered safe under current standards, based upon PTS [7–11]. Since the functional abnormalities related to the synaptic damage without PTS are not detectable by routine audiological evaluations, which are focused on thresholds, and may not be known to the subjects, they are umbrellaed under the term of noise-induced hidden hearing loss (NIHHL) [6, 8, 9, 12]. The synaptic damage and the resulted functional deficits seen in NIHHL are called synaptopathy. Since it can happen after a relatively low dose of noise exposure and before a PTS is established, it is likely more common than the NIHHL defined by a PTS. Therefore, noise-induced synaptopathy has become a new focus in the study of NIHL. Efforts have been made to develop an effective therapeutic method against this noise-induced synpatopathy [13–15].

Neurotrophin-3 (NT-3), a member of the neurotrophic factor family, is widely expressed in the cochlea during development; it is critical for the formation of IHC-SGN synapses and the survival of SGNs themselves [16–20]. Although restrictively expressed in IHCs and their adjacent supporting cells postnatally, NT-3 continues to play an important role in maintaining the survival of SGNs and their synapses to IHCs [21–23].

Increasing the level of NT-3 in the cochleae appears to be beneficial to protect the synapse from damage by noise. Exogenous NT-3 delivered to the round window just 24 h after moderate noise exposure promoted regeneration of both presynaptic and postsynaptic elements of ribbon synapses in the basal turn of the cochlea [24, 25]. The overexpression of NT-3 in supporting cells of transgenic mice could promote the recovery of the auditory response and the regeneration of ribbon synapses after noise damage [14]. However, direct application of exogenous NT-3 will not provide long-term protection; and the genetic modification at the genome level is not ethical and therefore not acceptable for human subjects.

Adeno-associated virus (AAV) is an ideal gene delivery vector because of its low immunogenicity and persistent gene expression in non-dividing cells [26]. The cochlea itself is an ideal organ for local gene therapy because of its isolation from surrounding tissues [27, 28]. We hypothesize that NT-3 overexpression mediated by AAV can effectively reduce the noise-induced damage to SGN–IHC synapses. In the present study, exogenous NT-3 was expressed in the cochlea by an AAV-mediated cochlear gene transfection before noise exposure. To evaluate the effectiveness of this approach in the protection against the noise-induced cochlear synaptopathy, a self-control design was used: in each animal one ear was given AAV for NT-3 overexpression, while the other ear was given saline as control. The protective effect was evaluated in far field by auditory brainstem response (ABR) and in near field by compound action potential (CAP). The functional evaluation was followed by the morphological analysis of cochlear synaptopathy. Here we demonstrated that AAV-NT-3 can promote the restoration of the synapses that were damaged by the noise and therefore reduced the consequences of NIHHL.

## Results

### NT-3 transfection efficiency in IHCs

**Fig. 1:**
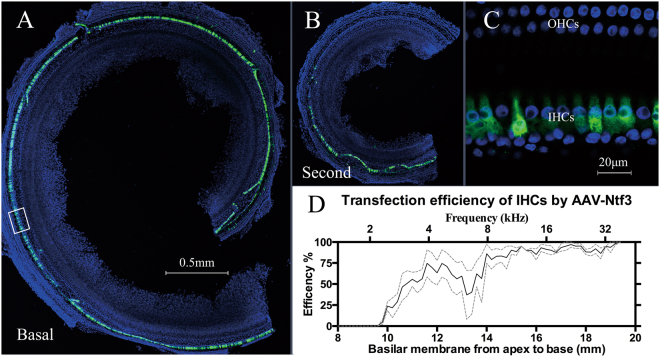
AAV-mediated NT-3 transfection in guinea pig cochleae. **a**, **b** Typical confocal images showing the transfection in the basal turn and second turn of the cochlea, stained with an anti-myc antibody (green) and DAPI (blue). **c** The higher magnification from the rectangle region of **a**, which represents the position of 16 kHz in the cochlea. **d** The averaged cochleogram (*n* = 3) showing the mean and ±SEM of transfected IHCs as a function of distance

The transfection of the AAV-mediated NT-3 in cochlear cells was demonstrated by the antibody against the myc tag (Fig. 1). The transfection is highly concentrated in IHCs, not to the adjacent supporting cells, with the viral vector used. A high-magnification image revealed that at least 80% IHCs was transfected in the basal turn (Fig. 1c). The transfection cochleogram showed a satisfactory transfection to IHCs from the basal end, up to the middle region of the cochlea (10 mm from the apex or 4 kHz region) (Fig. 1d), well covered the region of synaptic damage caused by noise exposure in the present study.

### Impact of noise exposure on hearing function and the protective effect of NT-3

**Fig. 2:**
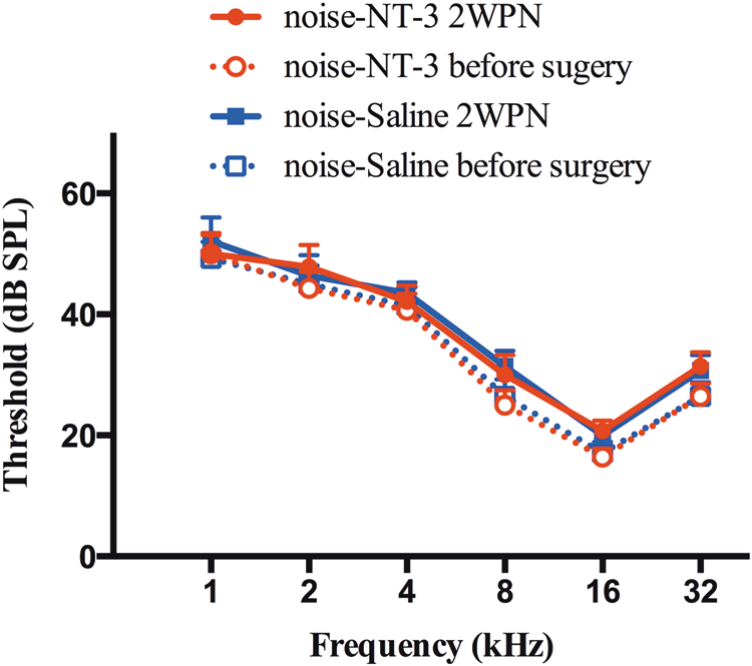
Tone-burst ABR audiograms. ABR threshold was measured before, 1 week after the transfection or the control surgery (not shown), and 2 weeks after the exposure to the 2 h noise exposure at 105 dB SPL. Thresholds shown are group means (±1 SEM) for seven ears tested per group. Two-way repeated-measurement ANOVA (group×frequency) did not show significant effect of noise exposure and surgery

ABRs were tested to examine the threshold of hearing three times: baseline test, 1 week after the transfection surgery, and 2 weeks after the noise exposure. A two-way repeated-measure (RM) analysis of variance (ANOVA) was performed against the factor of noise and surgery. No significant difference was seen between the baseline test and the one taken 1 week after the transfection surgery, suggesting the safety of the surgery for the injection of both AAV-NT-3 and saline via a cochleostomy. A high-pass noise with a cutoff at 4 kHz was given at 105 dB SPL for 2 h to create NIHHL. Two weeks after the noise exposure, ABR was repeated and the results showed no-significant threshold changes from the baseline test for both ears receiving NT-3 transfection or saline, respectively (Fig. 2).

**Fig. 3:**
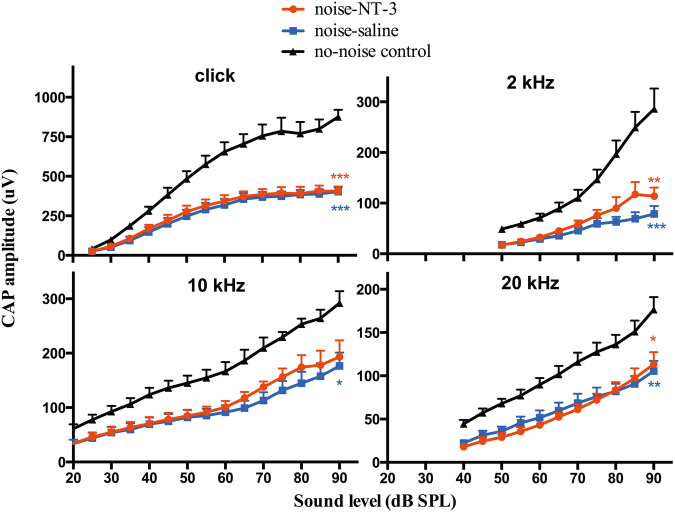
The CAP input/output function curves evoked clicks, and 2, 10, and 20 kHz tone bursts (*n* = 6 ears for each group). One-way ANOVA followed by Bonferroni’s post-hoc test was performed between groups at 90 dB SPL for each signal. Significant difference was seen between the no-noise control and those obtained from the ears exposed to the noise but not between the two ears exposed to the noise. Error bars represent ±1 SEM. The insert legends for individual curves applies to all panels. Asterisks indicates the significant level for the comparison between the two noise-exposed ears and the control. *p < 0.05, **p < 0.01, ***p < 0.001

The effect of noise on synaptic damage was evident by the amplitude reduction in the CAP after the noise exposure, which was measured in the input/output function by clicks and tone-bursts at 2, 10, and 20 kHz (Fig. 3). However, the input/output functions obtained after the noise exposure were largely overlapped between ears transfected with NT-3 and those in the saline control. One-way ANOVA followed by Bonferroni’s post-hoc test was performed across groups on the maximal CAP amplitudes that were obtained at the highest sound level tested (90 dB SPL). There was a significant difference between the no-noise control and the ears tested after the noise exposure in each of the signals tested. For the 10 kHz tone-burst, only the difference between the no-noise control and noise-saline ears was statistically significant. However, there was no significance between the ears receiving NT-3 gene transfection and those receiving saline.

**Fig. 4:**
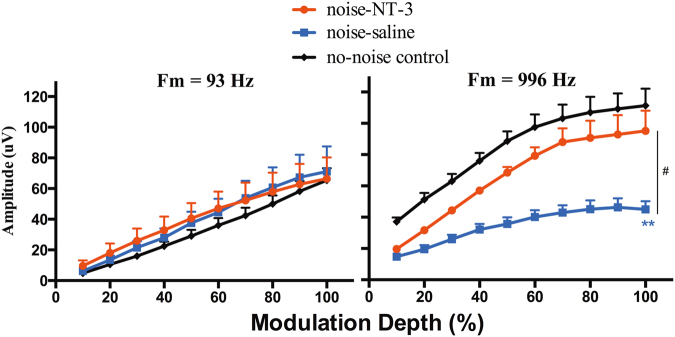
The comparison on AM CAP across groups (*n* = 6 ears for each group). The carrier frequency was 20 kHz. The AM CAP was tested with Fm at 93 Hz (**a**) and 996 Hz (**b**), respectively, to show the amplitude as the function of modulation depth in percentage. One-way ANOVA followed by Bonferroni’s post-hoc test was performed between groups at 100% modulation depth. The result showed that the AM CAP amplitude of the noise-saline group was significantly lower than that of the control group (**p < 0.01) as well as the value of the NT-3-noise group (^#^*p* < 0.05). Error bars represent ±1 SEM. The insert legends for groups applies to both panels

The cochlear function was further evaluated with CAP evoked by amplitude modulation (AM) of a 20 kHz tone. The modulation frequency (Fm) was 93 and 996 Hz, and the modulation depth varied from 10 to 100% in 10% steps to obtain AM depth curves (Fig. 4). While the AM CAP curves were largely overlapped with Fm = 93 Hz, they were separate across groups at Fm = 996 Hz and larger at larger modulation depths. At 100% modulation depth, the averaged AM CAPs were 111.3 ± 10.8, 95.2 ± 12.9, and 45.0 ± 5.27 μV, respectively, for the groups of no-noise control, noise-NT3, and noise-saline ears. One-way ANOVA on the AM CAP amplitude at 100% modulation depth showed a significant difference across groups (*F*_(2,15)_ = 11.503, *p* < 0.001). The Bonferroni’s post-hoc test showed that the AM CAP amplitude of he noise-NT-3 group was significantly higher than that of the noise-saline group (*t* = 3.84, *p* < 0.05). While the amplitude of the noise-saline group was significantly lower than that of the control group (*t* = 4.60, *p* < 0.01), there was no significant difference between the no-noise control and noise-NT-3 ears (*t* = 1.12, *p* = 0.847).

### Noise-induced change in synapse counts and the effect of NT-3 overexpression

**Fig. 5:**
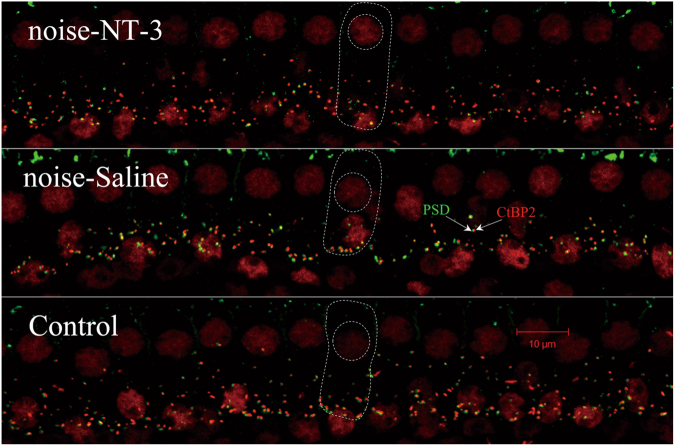
Representative immunostaining of presynaptic and postsynaptic structures across IHC-SGN synapses in the area of 22.6 kHz. The dash lines indicate the outline of IHCs and their nuclei. Red dots: puncta stained against CtBP2, green: puncta of PSD95

In this study, presynaptic and postsynaptic structures were double labeled with antibodies against the main presynaptic ribbon protein C-terminal binding protein 2 (CtBP2) and pos-synaptic densities 95 (PSD95), respectively, to show the change of synapse counts induced by the noise and the protective effect of NT-3. CtBP2 labeling stained both ribbons and nuclei, while PSD95 labeling also showed staining outside the synaptic regions. However, staining to synapses was disguisable by the location and the pairing between the presynaptic and postsynaptic components. The synapses were counted by the small red dots that were matched by the green dots of the similar size and were located at the bottom of IHCs. As compared to the control ears, the two groups of ears exposed to noise showed a significant reduction in the synapse counts examined 2 weeks after the noise exposure, mainly in the high-frequency region. Figure 5 shows the typical images of the 22.6 kHz region from these three groups, in which the presynaptic protein CtBP2 was stained red, while the PSD95 were green.

**Fig. 6:**
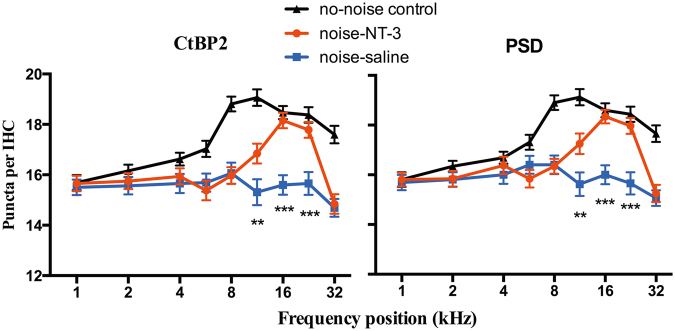
Comparison of synaptic density cochleograms across groups (*n* = 32 IHCs from seven cochleae of each group). The synaptic density was measured by counting the puncta of both CtBP2 (**a**) and PSD95 (**b**). The density cochleograms were made for the puncta/IHC as a function of frequency. The puncta were averaged over 32 IHCs obtained from 4 cochleae at each frequency point in each group. A two-way ANOVA followed by Bonferroni’s post-hoc test was performed to evaluate the density difference across groups at different frequencies. The synapse density of the noise-NT-3 group was found to be significantly higher than that of the noise-saline group at 11.3, 16, and 22.6 kHz (**p < 0.01, ***p < 0.001). Error bars represent ±1 SEM. The insert legend for the curves applies to all panels

Figure 6 compared the cochleograms across groups for both densities of presynaptic ribbon and PSD95 (no. per IHC) as a function to show the ribbon synapse loss after noise exposure and the protective effect of AAV-NT-3. An overall reduction in the density of CtBP2 and PSD95 was seen in both noise-exposed ears, mainly in the high-frequency region between 8 and 32 kHz. There was also an obvious difference between the two noise-exposed ears: much less reduction in the NT-3-transfected ears in the high-frequency region. A two-way ANOVA was performed against the grouping (main factor) and frequency (co-variant). The result showed a significant effect for both frequency (*F*_(8,837)_ = 12.93 and 13.46, respectively, for CtBP2/PSD, *p* < 0.001) and grouping (*F*_(2,837)_ = 76.84 and 66.57, respectively, for CtBP2/PSD, *p* < 0.001). Within the factor of frequency, significant differences were seen in the post-hoc tests (Bonferroni tests) at 11.3, 16, and 22.6 kHz. At 22.6 kHz, for example, the synapse densities counted by CtBP2 were 18.4 ± 0.3, 17.8 ± 0.3, and 15.7 ± 0.5 per IHC for the no-noise control, noise-NT-3, and noise-saline groups, respectively. The post-hoc tests showed that the CtBP2 density of the noise-NT-3 group was significantly higher than the noise-saline group (*t* = 4.32, *p* < 0.001), and there was no significant difference between the noise-NT-3 and the no-noise control groups.

## Discussion

In the present study, local overexpression of NT-3 was achieved by cochlear injection of an AAV carrying NT-3 DNA. An effective transfection was seen in the basal half of the cochlea, mainly in the IHCs, but not in the supporting cells. A brief noise exposure was used at a moderate level that did not produce a PTS but did produce massive damage to ribbon synapses between the IHCs and SGNs and that was biased to the high-frequency region. As tested 2 weeks after the noise exposure, the averaged synapse counts in the region between 11.3 and 22.6 kHz in the ears receiving saline was significantly lower than that of the control sample (Fig. 6) and the ears receiving NT-3 overexpression. Putting it all together, it is safe to say that more than half of the noise-induced synapse loss was reduced by the NT-3 overexpression in IHCs. This result is quantitatively comparable with the protection seen in a previous study in which NT-3 overexpression was achieved only in supporting cells using a transgenic mouse line [14]. Therefore, the overexpression of NT-3 in IHCs appears to be effective in synaptic protection. Further study is needed to see whether the overexpression in both IHCs and supporting cells will provide a better protection and whether the overexpression can cure synaptopathy after noise.

In the present study, we did not observe the loss of synapses immediately after the noise exposure. However, based upon the previous study in the transgenic mouse line, it is more likely that the NT-3 overexpression promoted the regeneration of the synapses, rather than protecting the synapses from direct noise damage [14]. The IHCs at 32 kHz had a robust expression of exogenous NT-3 (Fig. 1d). However, no improvement of synapse count was seen in this region (Fig. 6). We do not have a clear explanation for this discrepancy. A speculation is hinted by Wan’s previous study [14]: the recovery or synaptic repair in this region relies more on the NT3 from the supporting cells. Unfortunately, there is no data to support this speculation.

NT-3, but not brain-derived neurotrophic factor, is expressed in both hair cells and supporting cells in the postnatal cochleae [19]. Selective knockout of NT-3 in hair cells does not change the cochlear function (tested with the ABR threshold and wave I amplitude), while the selective overexpression of NT-3 in supporting cells resulted in a decrease in the ABR threshold and an increase in the wave I amplitude [14] and a regeneration of synapses after noise-induced damage [14].

The AAV-NT-3 we used in the present study resulted in a selective transfection in IHCs only but not in the supporting cells. In combination with the protective effect in the previous study with NT-3 overexpression in supporting cells only [14], it is recognized that NT-3 from both IHCs and supporting cells contributes to the synapse regeneration after damage by noise exposure. Since NT-3 overexpression in IHCs or in supporting cells provides comparable amount of partial restoration of synapses, it is interesting to see whether NT-3 overexpression in both IHCs and supporting cells will provide a better promoting effect on the synapse regeneration after noise damage. We are producing an AAV to ensure transfection to both hair cells and supporting cells for this purpose.

Tested with CAP I/O functions, we did not see any significant difference between the ears with NT-3 overexpression and the saline control before the noise exposure. This appears to be different from the increased ABR wave I reported by Wan et al. [14] in mice with NT-3 overexpression. In this study, the increased response amplitude and decreased ABR threshold were accompanied by an increased synapse density in subjects with NT-3 knock-in in the supporting cells. However, such results were not seen in the subjects with the knock-in limited to IHCs. This suggests that the overexpression of NT-3 in the supporting cells is more powerful in IHC–SGN synapse formation. In addition, Wan’s study was in mice with conditional knock-in of NT-3. Potentially, there is a species difference. Although no data are available for a comparison, the overexpression by the gene knock-in is likely to be stronger than that mediated by AAV.

The results of I/O functions also seemed to conflict with our morphological data. However, it is understandable considering the fact that the noise-induced synaptic damage is biased to the synapses that innervate auditory nerve fibers with low spontaneous spike rate (SR) [5, 6, 29], and those fibers make less contribution to CAP, which is the onset response to the sound [30].

Consistent with the fact that the noise-induced synaptopathy is biased to the synapses innervating the auditory nerve fibers with low SR, the CAP evoked by AM at the high sound level (80 dB SPL) reduced the amplitude significantly after the noise exposure. It is worth to notice three aspects indicated by the data obtained in this study. First is the fact that the reduction is significant only at the higher modulation frequency (996 Hz) examined, suggesting that the auditory synchrony to AM is more challenging at this high Fm frequencies and relies more on the low SR auditory nerve fibers. This is consistent with a recent study that examined noise-induced changes in the AM-evoked far-field response [31]. In this study using CBA mice, the biggest reduction in the scalp-recorded AM responses induced by noise damage was found with a Fm close to 1 kHz. Our data support that the far-field recording of AM responses is useful for reflecting the change of cochlear synchrony to AM. The second aspect is the fact that the difference in AM CAP amplitude between no-noise control and the noise-exposed ears is larger at deeper modulation depth. This is conflicted with the suggestion from previous reports that AM with shallow modulation depths may be more sensitive to diagnose the supra-threshold coding deficits [32, 33]. Our data suggest that AM signals with deeper modulations produce larger ranges of intensity change and therefore rely more on the low SR auditory nerve fibers. The third aspect is the less reduction of AM CAP amplitude in the ears with NT-3 overexpression, which is the functional evidence for the protection, consistent with the morphological data.

In conclusion, the present study proved that NT-3 overexpression, established by an AAV-mediated cochlear gene transfection, could provide effective protection against noise-induced damage to the IHC–SGN synapses innervating the low-SR nerve fibers. We think that this treatment has the potential to be translated into human subjects to prevent or cure the synaptopathy by noise. However, there is a long way to go before this goal is to be realized. Further studies are needed to verify and improve the effectiveness of this treatment in (1) preventing the synaptopathy by long term or repeated noise exposure, and in (2) repairing synapse after noise insult. Further studies are also needed to reduce the risk of the cochlear gene transfection. The therapy will only be acceptable by subjects with no hearing loss when the benefit is superior largely over the cost/risk.

## Materials and methods

### Subjects and general procedures

In total, 17 male albino adult guinea pigs (2–3 months old) were recruited for this study from the Experimental Animal Service of Medical School, Shanghai Jiao Tong University. Out of the 17 guinea pigs, 3 were used for evaluating the transfection rate of the virus, 7 were for evaluating the NT-3 protection against noise (noise group), and 7 as no-noise controls. In the seven subjects used in the noise group, each animal received AAV-NT-3 transfection in one ear that was randomly selected and the saline in the other ear. The sample size was determined with a power analysis based on the data distribution of noise-induced synapse count change in our previous studies [1, 5, 34]. Therefore, in terms of the ears examined, there were three groups that were named as control, noise-NT-3, and noise-saline, respectively (*n* = 7 in each). A baseline ABR test was performed in closed field to ensure good hearing of each animal. Four days after the baseline ABR, transfection surgery was done on animals in the experimental group. One week after the surgery, ABR was repeated in this group to verify the threshold shift caused by the surgery. Then the animals were exposed to the noise to establish cochlear synaptopathy. Two weeks after the noise exposure, the animals in both the noise group were examined in an end test of CAP in near-field recording, followed by morphological observations. The control animals were also examined in the same way at the same age. Four cochleae in each group were used for the ribbon synapse count. All the procedures were approved for ethics by the Institutional Animal Care and Use Committee of Shanghai Sixth People’s Hospital affiliated to Shanghai Jiaotong Unversity (permit number 2016-0327). The timeline and the major procedures of this study were graphically summarized in supplemental Fig. 1.

### Surgery

Cochleostomy was performed for AAV/saline injection in subjects that were anesthetized with inhalant isoflurane (4% for induction, 2% for maintenance, 0.3 L/min O_2_ flow rate). The animal was placed in the lateral position on a thermostatic heating pad to maintain the body temperature at 38 °C. Approximately, 0.3 mL 1% lidocaine was injected subcutaneously in the postauricular area for local anesthesia, where a 2-cm arc incision was made along the root of the earlobe. The posterior part of the auditory bulla was exposed by blunt dissection. A hole of 2 mm diameter was made on the bulla to expose the basal turn of the cochlea and the round window niche. A small hole of 0.3 mm diameter was made through the bony wall of the scala tympani of the basal turn of the cochlea. Cochlear perilymph injection was done using a glass tip made from 34 G microfilm that was connected to a microsyringe pump (Micro4; WPI, Kissimmee, USA) via a polyethylene tube. Also, 10 μL of viral vector/saline was injected at the rate of 20 nL/s into the scala tympani. The selection of ears receiving either AAV or saline was randomized but not blinded. The hole of cochleostomy was sealed with muscle tissue, and the hole of bulla was closed by suture of muscle and skin incision. The surgery on one side took about 30 min. The AAV-NT-3 vector was serotype 8 AAV with surface tyrosine mutation at 733 amino acid on the viral capsid (rAAV8-mut733). A cDNA expression construct encoding the full length of the guinea pig NT-3 cDNA open reading frame with an N-terminal myc tag was constructed in pTR vector under the control of chicken beta-actin promoter. The vector was provided by the Retinal Gene Therapy Group, University of Florida, USA at the titers of 6.92 × 10^13^.

### Noise exposure

The animals were placed in a metal-wire cage, awake and unrestrained, during the noise exposure. Electrical Gaussian noise was generated and high-pass filtered with a cutoff at 4 kHz by RP2 signal processor from Tucker-Davis Technologies (TDT System III; Alachua, FL, USA). The output of the signal processor was amplified by an audio-amplifier (Yamaha PS9500, Shenzhen, China) and delivered to a four-speaker array (Pyramid TW-67 tweeters, Amazon.com) suspended 40 cm above the animals. The exposure was given at 105 dB SPL for 2 h. The acoustic spectrum of the noise was distributed mainly between 4 and 22 kHz (supplemental Fig. 2). The noise level was monitored using a 1/4-inch microphone linked to the RP2 module of the TDT system through which the sound level was calculated by a RPvdx circuit.

### Physiological tests

The animals were anesthetized with ketamine mixed with xylazine (40 mg/kg and 4 mg/kg, i.p.) and the body temperature was maintained at 38 °C with a thermostatic heating pad. For the ABR test, the acoustic signals were delivered via plastic tubing to the tested ear (closed field); the responses were recorded with three subdermal electrodes with the recording electrode inserted at the vertex and the reference and grounding electrodes posterior to the external auditory canals. CAP was recorded via a silver-wire electrode that was placed on the round window membrane. The electrode was made from Teflon-coated silver wire (0.005 inch in diameter, cat. # 78600, A-M System Inc.). The insulation was cut off by 2 mm at the tip and the naked wire was coiled to make a ring. The surgery was done to expose the round window membrane and to place the recording electrode. The reference and grounding electrodes were inserted into the muscle surrounding the incision. The other ends of the three electrodes were led to a RA16PA preamplifier.

Hardware and software from Tucker-Davis Technologies (TDT System III; Alachua, FL, USA) were used for stimuli generation and bio-signals' acquisition. The acoustic stimuli used were as follows: (1) clicks for CAP (0.1-ms duration, presented at the rate of 21.1/s), (2) tone bursts for ABR and CAP (10-ms duration with cos2 gating and 0.5-ms rise/fall time, at the rate of 21.1/s), and (3) 20 kHz AM tones for CAP (500-ms duration with rise/fall time 5 ms, at the rate of 1.5/s). The stimuli for the ABR were played out through a broadband speaker (MF1; TDT) and delivered to the tested ear via a 10 cm tubing. The signals for the CAP was delivered via another broadband speaker (Fostex FT28D Dome Tweeter, Madisound, USA) that was placed 10 cm in front of the animal. For ABR tests and CAP I/O functions, the sound level was decreased in 5-dB steps from 90 dB SPL until the response disappeared. For AM CAPs, the sound was presented at 80 dB SPL with Fms at 93 and 996 Hz, respectively. The evoked responses were amplified 20× by a PA16 preamplifier (TDT) and filtered between 100 and 3000 Hz for click and tone-burst ABRs or between 10 and 3000 Hz for AM CAPs. The responses were averaged 1000 for the ABRs and 100 for the CAPs. The ABR thresholds were tested at 1, 2, 4, 8, 16, and 32 kHz, defined as the lowest level where a repeatable wave III response was observed. The AM CAP was measured by detecting the peak of the modulation frequency in the spectrum analysis of the averaged 500 ms sweep after the first and the last 50 ms of responses were cut off. The data from one ear in each group were excluded due to the instability of CAP during the test.

### Morphology

Following the endpoint physiological tests, the animals were sacrificed with an overdose of pentobarbital (100 mg/kg, i.p.), and the cochleae were harvested. The cochleae were quickly harvested and perfused rapidly with 4% paraformaldehyde in phosphate-buffered saline (PBS) and immersed in paraformaldehyde for 1 h fixation at 4 °C. Then each cochlea was transferred into PBS, and the bony shell of the cochlea was removed with a fine forcep. After removing the tectorial membrane, it was permeabilized with 1% Triton X-100 in PBS for 60 min and then incubated in 5% goat serum in PBS for another 60 min. The cochlea tissue, used for evaluation of the transfection efficiency, was incubated with the primary antibody rabbit anti-myc (IgG; ABclonal, cat. AE009, 1:200). The tissue from other cochleae used for synapse counts were incubated with the mixture of mouse anti-CtBP2 antibody (IgG1; BD Biosciences, cat. # 612044, 1:200) and mouse anti-PSD95 antibody (IgG2a; Millipore, cat. # MAB1596, 1:600). After storage overnight at 4 °C, the PBS-washed cochleae were treated with corresponding secondary antibodies (goat anti-rabbit IgG, goat anti-mouse IgG1 and IgG2a, 1:800, Invitrogen A11034, A21124, and A21131, respectively) for 2 h at room temperature. After immunostaining, the cochleae were post-fixed with paraformaldehyde again for 60 min. The basilar membranes were dissected into four pieces and mounted on the microscopic slides. The basilar membrane for transfection efficiency evaluation was further counterstained with 4,6-diamidino-2-phenylindole (DAPI; Fluoroshield with DAPI; Sigma-Aldrich, cat. # F6057) and cover slipped. Confocal images at the specific frequency position (1, 2, 4, 5.6, 8, 11.3, 16, 22.6, and 32 kHz) were acquired using a confocal laser-scanning microscope (LSM 710 META; Zeiss, Shanghai, China) with the 63× water-immersion objective. Image stacks were then exported to the image-processing software, ImageJ (NIH), and eight successive IHCs at each frequency position of the cochleae were selected to count their puncta of CtBP2 (in red) and PSD95 (in green).

### Statistics

All data are expressed as means ± standard error of the mean (SEM). Statistics was performed using the statistical package for the social sciences (SPSS) software (ver. 23; SPSS Inc., Chicago, IL). A difference was considered statistically significant if *p* < 0.05.

## Electronic supplementary material

Supplemental Figure 1. Schematic time-line of the experiment

Supplemental Figure 2. The power spectrum of the noise
