# Supplementary figures and images for: AAV-mediated NT-3 overexpression protects cochleae against noise-induced synaptopathy
Source: Gene Ther. 2018 Mar 13;25(4):251–9. doi: 10.1038/s41434-018-0012-0 (PMC6062503; doi:10.1038/s41434-018-0012-0)

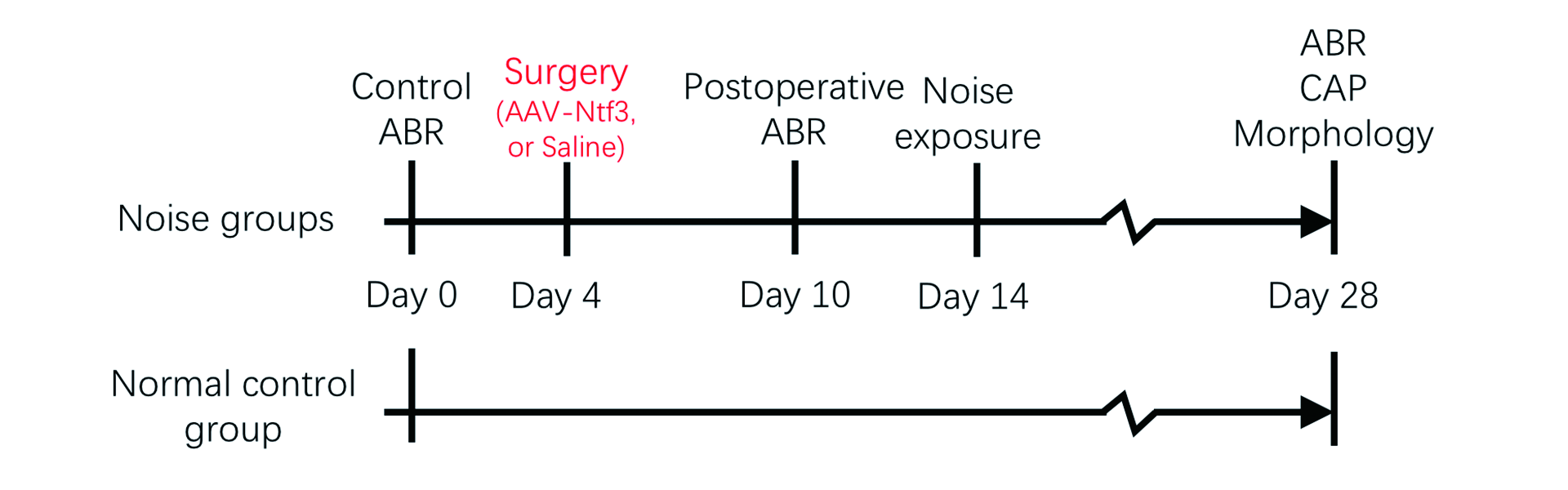

Supplement: Supplementary file 1 — Supplemental Figure 1. Schematic time-line of the experiment [file 41434_2018_12_MOESM1_ESM.tif]

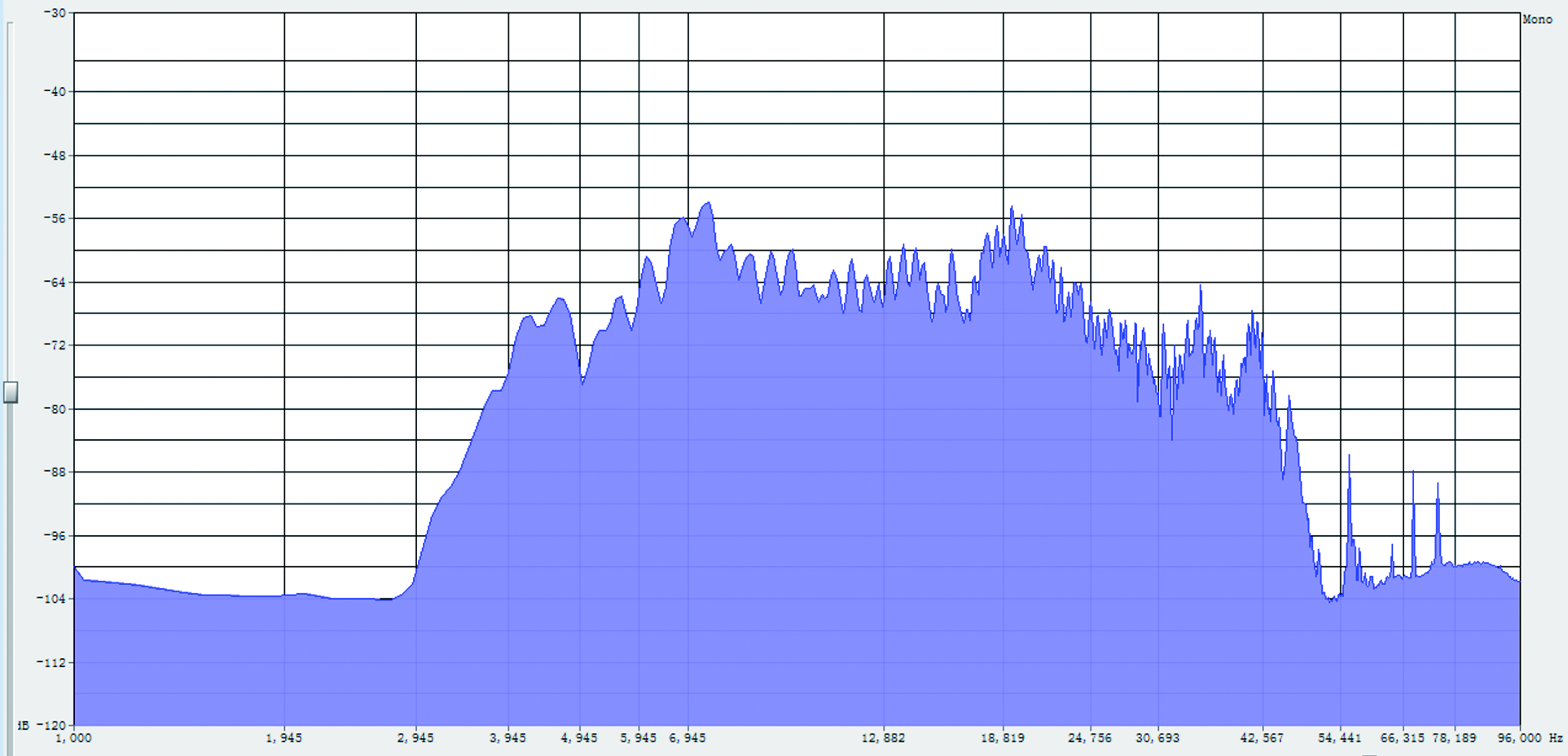

Supplement: Supplementary file 2 — Supplemental Figure 2. The power spectrum of the noise [file 41434_2018_12_MOESM2_ESM.tif]
